# A New Fitness Test of Estimating VO_2max_ in Well-Trained Rowing Athletes

**DOI:** 10.3389/fphys.2021.701541

**Published:** 2021-07-02

**Authors:** Wei Dong Gao, Olli-Pekka Nuuttila, Hai Bo Fang, Qian Chen, Xi Chen

**Affiliations:** ^1^Zhejiang Institute of Sports Science, Hangzhou, China; ^2^School of Sports Science, Wenzhou Medical University, Wenzhou, China; ^3^Faculty of Sport and Health Sciences, University of Jyväskylä, Jyväskylä, Finland

**Keywords:** maximal aerobic capacity, flatwater rowers, flatwater paddlers, submaximal fitness test, treadmill running, rowing

## Abstract

**Background:**

This study was designed to investigate the validity of maximal oxygen consumption (VO_2max_) estimation through the Firstbeat fitness test (FFT) method when using submaximal rowing and running programs for well-trained athletes.

**Methods:**

Well-trained flatwater rowers (*n* = 45, 19.8 ± 3.0 years, 184 ± 8.7 cm, 76 ± 12.9 kg, and 58.7 ± 6.0 mL⋅kg^–1^⋅min^–1^) and paddlers (*n* = 45, 19.0 ± 2.5 years, 180 ± 7.7 cm, 74 ± 9.4 kg, and 59.9 ± 4.8 mL⋅kg^–1^⋅min^–1^) completed the FFT and maximal graded exercise test (GXT) programs of rowing and running, respectively. The estimated VO_2max_ was calculated using the FFT system, and the measured VO_2max_ was obtained from the GXT programs. Differences between the estimated and measured VO_2max_ values were analyzed to assess the accuracy and agreement of the predictions. Equations from the previous study were also used to predict the VO_2max_ in the submaximal programs to compare the accuracy of prediction with the FFT method.

**Results:**

The FFT method was in good agreement with the measured VO_2max_ in both groups based on the intraclass correlation coefficients (>0.8). Additionally, the FFT method had considerable accuracy in VO_2max_ estimation as the mean absolute percentage error (≤5.0%) and mean absolute error (<3.0 mL⋅kg^–1^⋅min^–1^) were fairly low. Furthermore, the FFT method seemed more accurate in the estimation of VO_2max_ than previously reported equations, especially in the rowing test program.

**Conclusion:**

This study revealed that the FFT method provides a considerably accurate estimation of VO_2max_ in well-trained athletes.

## Introduction

Maximal oxygen consumption (VO_2max_) is defined as the maximal capacity of the pulmonary, cardiovascular, and muscular systems to deliver and utilize oxygen, which can reflect an individual’s cardiorespiratory fitness (Saltin and Strange, 1992; Bassett and Howley, 1997; Levine, 2008). Measurement of VO_2max_ provides important outcomes for both physical performance and health status in general (Brink-Elfegoun et al., 2007), and it is frequently used to assess the aerobic capacity of professional or amateur athletes (Shephard, 2009). Therefore, VO_2max_ is often used in endurance sports to provide training and athletes’ performance information to coaches (Midgley et al., 2009; Sartor et al., 2013).

VO_2max_ can be measured through direct methods with a metabolic gas measurement system, with the athlete performing a maximal graded exercise test (GXT) until exhaustion. This is regarded as the gold standard as it can obtain an accurate value of VO_2max_ (Beltz et al., 2016). However, as it is time-consuming and there is an economic burden, the use of direct measurement methods is limited. In addition, the exhausting exercise program affects the training arrangement of the season (Montgomery et al., 2009; Tanner and Gore, 2013; Riebe et al., 2018). From this perspective, indirect methods of estimating VO_2max_ based on the submaximal exercise program seem to be a good choice for athletes or teams, and these can frequently be used during the training season.

Previous studies have reported several indirect methods for estimating VO_2max_ in athletes based on running programs (Tsiaras et al., 2010; Marsh, 2012; Matabuena et al., 2018). Marsh (2012) found a four-stage incremental running program estimating VO_2max_ well, and the equation was fairly accurate [standard error of estimate (SEE) = 3.98-4.08 mL⋅kg^–1^⋅min^–1^; *r* = 0.642-0.646], as recommended by the American College of Sports Medicine. However, this equation is more suitable for males than for females, as the correlation data were conducted in male athletes. Other studies also reported submaximal VO_2max_ predictive equations [(SEE) = 2.52–3.51 mL⋅kg^–1^⋅min^–1^; *r* = 0.85–0.91] for the amateur exercise population (Larsen et al., 2002; Vehrs et al., 2007).

Klusiewicz et al. (2016) developed a predictive equation (*r* = 0.711) based on the PWC170 obtained from a submaximal rowing program (Klusiewicz and Faff, 2003), which was later validated to assess the aerobic fitness of rowers. The equation based on the rowing programs had not reached an accuracy similar to that from the equation based on the running programs, with correlation coefficients of 0.55 in male rowers (Klusiewicz and Faff, 2003) and *R*^2^ values of 0.79 and 0.64 in male and female rowers, respectively, Klusiewicz et al. (2016). Thus, the present study was designed to develop a new indirect method of VO_2max_ estimation for both male and female rowing athletes.

Recently, a new system [Firstbeat fitness test (FFT)] was used for the indirect estimation of VO_2max_. The estimated VO_2max_ value is automatically generated after collecting heart rate (HR) data from a configurable test program (rowing or running) using Firstbeat sports software (Firstbeat, Jyvaskyla, Finland). Studies have revealed that the Firstbeat VO_2max_ estimation system is valid in nonathletic populations (Kraft and Roberts, 2017; Kyröläinen et al., 2018; Anderson et al., 2019; Passler et al., 2019). To the best of our knowledge, no study has reported the accuracy of this system in estimating VO_2max_ in well-trained athletes. Therefore, this study was designed to investigate the accuracy of VO_2max_ estimation based on the FFT system when using submaximal rowing and running programs. In addition, this study aimed to evaluate the cross-validation of previous VO_2max_ predictive equations in both submaximal running (Marsh, 2012) and rowing programs (Klusiewicz et al., 2016), providing more information on the VO_2max_ estimated by the FFT system to coaches and sports scientists. The hypothesis for this study was that the FFT method could provide accurate VO_2max_ estimation in well-trained athletes, which would varify a new accurate option for estimating VO_2max_ in athletic population.

## Materials and Methods

The FFT system was used to estimate the participants’ VO_2max_ values, which were also compared to the VO_2max_ values from the direct method measurement of the GXT programs. Additionally, a cross-validation design was used to evaluate the validation of the VO_2max_ estimation when compared to other classical predictive equations based on submaximal rowing (Klusiewicz et al., 2016) and running programs (Marsh, 2012).

### Participants

A total of 90 well-trained athletes were recruited from Zhejiang Water Sports Training Center and divided into two groups based on the sports items (i.e., rowers and paddlers): 45 flatwater rowers (23 males and 22 females) in the ROW group and 45 flatwater paddlers (29 males and 16 females) in the RUN group (participant characteristics are shown in Table 1). This study was conducted according to the guidelines in the Declaration of Helsinki and was approved by the ethical committee of Zhejiang Institute of Sports Science (ZJSS201909162). All participants were informed of the details of the entire program and signed an approved informed consent document.

**TABLE 1:** Descriptive characteristics of the participants.

	**ROW group**			**RUN group**		
	**Male**	**Female**	**All**	**Male**	**Female**	**All**
	**(*n* = 23)**	**(*n* = 22)**	**(*n* = 45)**	**(*n* = 29)**	**(*n* = 16)**	**(*n* = 45)**
Age (years)	20.7 ± 3.6	19.0 ± 2.1	19.8 ± 3.0	18.7 ± 2.3	19.5 ± 3.0	19.0 ± 2.5
Height (cm)	190.7 ± 5.4	176.6 ± 4.7	184 ± 8.7	184.0 ± 4.6	171.4 ± 5.0	180 ± 7.7
Body mass (kg)	84.5 ± 3.6	67.0 ± 8.9	76 ± 12.9	78.7 ± 7.0	65.7 ± 7.0	74 ± 9.4
Training experience (years)	4.7 ± 3.1	3.9 ± 2.1	4.3 ± 2.6	3.7 ± 1.9	4.6 ± 2.2	4.0 ± 2.1

### Procedures

**FIGURE 1:**
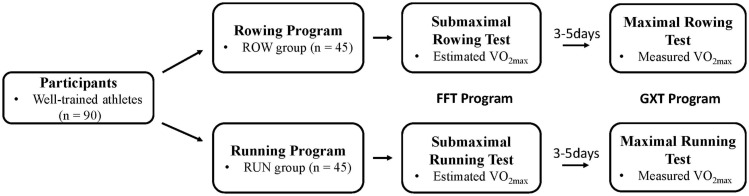
Experimental tests diagram.

Figure 1 shows the flow diagram of this study. First, submaximal FFT programs were performed, the ROW group athletes performed a submaximal incremental rowing test and the RUN group athletes performed a submaximal running test. Every athlete’s HR (Firstbeat, Jyvaskyla, Finland) was collected during the programs, and the estimated VO_2max_ value was obtained from FFT program. After 3–5 days of recovery from the submaximal program, the participants performed a maximal graded rowing (ROW group athletes) or running program (RUN group athletes) to obtain the measuredVO_2max_ value using a breath-by-breath metabolic measurement system, which was regarded as the golden standard test of VO_2max_ (Fletcher et al., 2013). All experimental tests were performed at the same time frame during the regular training period (9:00–12:00, 15:00–18:00) in a quiet and air-conditioned laboratory (temperature, 18–23°C; humidity, 40–60%). The participants were asked to avoid heavy load training 7 days before the tests and during the recovery days, as well as abstain from caffeine and alcohol for 24 h before testing.

### FFT Program

The ROW group athletes performed a submaximal incremental rowing test on the rowing ergometer (model E, Concept 2, Morrisville, VT, United States), while the RUN group athletes performed a submaximal running test on the treadmill (H/P/Cosmos, Nussdorf, Germany). According to previous studies (Marsh, 2012; Klusiewicz et al., 2016), a multistage incremental test program with up to 85% maximal HR (HR_max_) was suggested to provide reliable predicted VO_2max_. To determine the appropriate intensity for the submaximal programs, a pilot study of 10 athletes (five rowers and five paddlers) was conducted to obtain the physiological responses for different stages, especially the last stage on the rowing ergometer or running treadmill. The submaximal incremental exercise test program consisting of four 3-min rowing exercises (an initial workload of 160 W in male athletes and 120 W in female athletes) and treadmill running (an initial running speed of 9 km/h in male athletes and 8 km/h in female athletes) was designed based on the pilot study and then performed by the ROW and RUN group athletes, respectively. Detailed information on the two programs is shown in Supplementary Table 1. The Firstbeat HR chest belt was used to record the HR during the entire test program. All rowers completed FFT rowing program and paddlers completed FFT running program. Finally, the Firstbeat Sports software (version 4.7.3.1, Jyvaskyla, Finland) automatically produced estimated VO_2max_ values based on the collected HR data of the submaximal FFT program.

### GXT Program

The ROW group athletes performed a maximal incremental rowing test program (Tanner and Gore, 2013) on the Concept II rowing ergometer, with an initial workload of 160 W in males and 120 W in females. The RUN group athletes performed the Bruce running program on a treadmill (Riebe et al., 2018). Detailed information on the two maximal programs is shown in Supplementary Table 2. A breath-by-breath metabolic measurement system (Quark PFT Ergo, Cosmed, Rome, Italy) was used to record the respiratory gas information throughout the whole GXT procedure in both the ROW and RUN groups. The system was calibrated in advance by following the manufacturer’s instructions. During the testing process, the rating of perceived exertion (RPE) on the Borg scale (6–20) and the HR (SZ990, Cosmed, Rome, Italy) were also recorded at the end of each stage manually. The achievement of VO_2max_ was identified when meeting at least two of the three following criteria (Howley et al., 1995): (1) achievement of the oxygen consumption plateau with an increasing workload, (2) respiratory exchange ratio (RER) reached ≥1.10, and (3) the HR reaching within 10 beats of the age-adjusted HR_max_ upon using the equation, 220 - age. The value of the measured VO_2max_ was defined as the highest 30-s average value of VO_2_ measured during GXT (Midgley et al., 2007).

### Statistical Analysis

All data were presented as mean ± standard deviation. The Shapiro–Wilk test was performed to test the normality of the outcome variables. Then Pearson’s correlation between the estimated VO_2max_ values from the submaximal test programs and the measured VO_2max_ values from the direct method using the GXT program was performed to assess the correlation magnitude and coefficient of determination (*R*^2^). To assess the accuracy of the estimation, the mean absolute percentage error (MAPE) and mean absolute error were calculated. The intraclass correlation coefficient (ICC) was used to determine the agreement between the estimated VO_2max_ values and measured VO_2max_ values. Furthermore, the Bland-Altman plot was used to investigate the level of agreement with the 95% limits of agreement (Bland and Altman, 1986). All statistical analyses were performed using IBM SPSS Statistics software for Windows (version 24 IBM, Armonk, NY, United States), and statistical significance was set at *P* < 0.05.

## Results

### Accuracy of VO_2max_ Estimation From FFT

**FIGURE 2:**
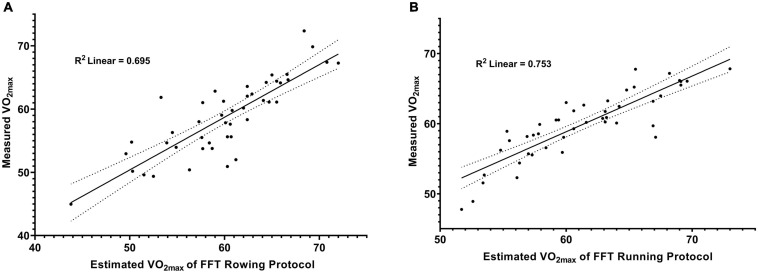
The linear regression plots between the estimated VO_2max_ from FFT and measured VO_2max_ by GXT. **(A)** The FFT rowing program and **(B)** The FFT running program. The coefficient of determination (*R*^2^) and 95% confidence interval bounds (dotted line) are also depicted.

Table 2 shows the VO_2max_ values measured by GXT programs and the HR, RPE, and RER in the last stage of the GXT program, and Table 3 shows the estimated VO_2max_ from the FFT and the analysis of correlations and differences between the two VO_2max_ values. The results showed that the estimated VO_2max_ was significantly overestimated in both ROW [constant error (CE) = 1.3 ± 3.5 mL⋅kg^–1^⋅min^–1^, *t* [44] = 2.501, and *P* = 0.016] and RUN (CE = 1.4 ± 2.6 mL⋅kg^–1^⋅min^–1^, *t* [44] = 3.624, and *P* < 0.001) submaximal test programs (Table 3). However, the results of the ICC revealed that the estimated VO_2max_ from the FFT had a good level of agreement with the directly measured VO_2max_ from the GXT in both the ROW (0.818, *P* < 0.001) and RUN groups (0.834, *P* < 0.001). Additionally, the results also showed a fairly low MAPE in both the ROW and RUN groups (ROW, 5.0%; RUN, 4.1%), which also verified that the FFT was considerably accurate in VO_2max_ estimation. Furthermore, linear regression plots demonstrated a good predictive model of the FFT method in both the rowing (*R*^2^ = 0.695, *P* < 0.001, and SEE = 3.35 mL⋅kg^–1^⋅min^–1^; Figure 2A) and running submaximal programs (*R*^2^ = 0.753, *P* < 0.001, and SEE = 2.43 mL⋅kg^–1^⋅min^–1^; Figure 2B).

**TABLE 2:** Results of the maximal graded exercise test.

	**ROW group**			**RUN group**		
	**Male**	**Female**	**All**	**Male**	**Female**	**All**
	**(*n* = 23)**	**(*n* = 22)**	**(*n* = 45)**	**(*n* = 29)**	**(*n* = 16)**	**(*n* = 45)**
HR at VO_2max_ (bpm)	189.0 ± 4.5	190.8 ± 5.3	189.9 ± 4.9	198.0 ± 5.2	193.3 ± 8.5	196.3 ± 6.9
RPE at VO_2max_ (6–20)	18.2 ± 1.1	18.2 ± 0.9	18.2 ± 1.0	18.9 ± 0.8	18.8 ± 1.1	18.9 ± 0.9
RER at VO_2max_	1.13 ± 0.1	1.11 ± 0.1	1.12 ± 0.1	1.13 ± 0.1	1.08 ± 0.1	1.11 ± 0.1
VO_2max_ (mL⋅kg^–1^⋅min^–1^)	60.7 ± 5.9	56.7 ± 5.5	58.7 ± 6.0	62.3 ± 3.4	55.6 ± 4.0	59.9 ± 4.8

*HR, heart rate; RPE, rating of perceived exertion on Borg scale 6–20; RER, respiratory exchange ratio; and VO_2max_, maximal oxygen consumption.*

**TABLE 3:** The correlations and differences between the estimated VO_2max_ from FFT and the measured VO_2max_ from GXT.

	**Gender**	**Estimated VO_2max_**	**Measured VO_2max_**	**CE**	***t***	***r***	**ICC**	**MAE**	**MAPE (%)**
		**(mL⋅kg^–1^⋅min^–1^)**	**(mL⋅kg^–1^⋅min^–1^)**	**(mL⋅kg^–1^⋅min^–1^)**				**(mL⋅kg^–1^⋅min^–1^)**	
**ROW**									
	Male (*n* = 23)	62.5 ± 6.0	60.7 ± 5.9	1.8 ± 3.6	2.429*	0.798*	0.736*	3.0 ± 2.6	5.3
	Female (*n* = 22)	57.5 ± 6.3	56.7 ± 5.5	0.7 ± 3.3	1.047	0.851*	0.841*	2.7 ± 2.0	4.8
	All (*n* = 45)	60.0 ± 6.0	58.7 ± 6.0	1.3 ± 3.5	2.501*	0.834*	0.818*	2.9 ± 2.3	5.0
**RUN**									
	Male (*n* = 29)	63.2 ± 4.8	62.3 ± 3.4	0.9 ± 2.6	1.838	0.851*	0.787*	2.2 ± 1.6	3.5
	Female (*n* = 16)	58.0 ± 4.4	55.6 ± 4.0	2.4 ± 2.4	3.902*	0.837*	0.727*	2.7 ± 2.0	5.0
	All (*n* = 45)	61.3 ± 5.3	59.9 ± 4.8	1.4 ± 2.6	3.624*	0.868*	0.834*	2.4 ± 1.7	4.1

CE, constant error, arithmetic mean of the difference between estimated and measured VO_2max_; *t*, *t* value from paried sample *t*-test; *r*, pearson correlation coefficient; ICC, intraclass correlation coefficient; MAE, mean absolute error; and MAPE, mean absolute percentage error. *Statistically significant (*P* ≤ 0.05).

### Level of Agreement Between the Estimated VO_2max_ From the FFT and the Directly Measured VO_2max_ From the GXT Program

**FIGURE 3:**
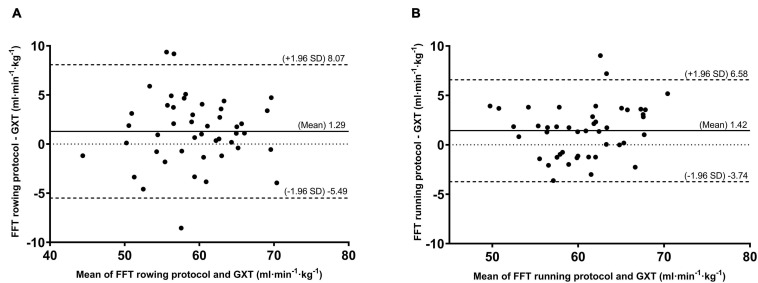
Bland-Altman plots between the estimated VO_2max_ from FFT and measured VO_2max_ by GXT. **(A)** The FFT rowing program and **(B)** The FFT running program. Mean biases (solid line), ±95% limits of agreement (dashed line), and equality (dotted line) are also depicted.

The Bland-Altman plots also demonstrated an agreement between the estimated VO_2max_ from the FFT and the directly measured VO_2max_ from the GXT program (Figure 3). The findings revealed that the estimated VO_2max_ had fairly low mean differences (bias) in both the ROW (Figure 3A) and RUN (Figure 3B) groups (bias: 1.29 mL⋅kg^–1^⋅min^–1^ for rowing and 1.42 mL⋅kg^–1^⋅min^–1^ for running). Furthermore, the FFT rowing program had a larger range of bias than that in the running program when estimating VO_2max_ from the FFT [upper to lower limits of agreement (ULoA-LLoA): 13.56 mL⋅kg^–1^⋅min^–1^ vs. 10.32 mL⋅kg^–1^⋅min^–1^, respectively].

### Comparison Between the FFT Method and Previous Predictive Equations

We then examined previous equations that predict VO_2max_ based on Klusiewicz et al.’s (2016) and Marsh’s (2012) studies using the HR and PWC170 (the equations and the predicted VO_2max_ are shown in Table 4) and compared them to the directly measured VO_2max_ from the GXT program. It was found that although Eq. 1 had a fairly accurate prediction of VO_2max_ in the rowing program, the FFT method had better accuracy and lower error terms for the overall ROW group (CE = 2.9 ± 2.3 mL⋅kg^–1^⋅min^–1^ and ICC = 0.818), as well as in both male (CE = 3.0 ± 2.6 mL⋅kg^–1^⋅min^–1^ and ICC = 0.736) and female (CE = 2.7 ± 2.0 mL⋅kg^–1^⋅min^–1^ and ICC = 0.841) subgroups. In addition, in the RUN group, Eqs 2, 3 had similar validity coefficients (*r* = 0.655 and 0.671, respectively) and level of agreement (ICC = 0.615 and 0.586, respectively) when the athletes performed the running program. However, in the female subgroup, neither Eq. 2 nor 3 showed a significant correlation with directly measured VO_2max_ from the GXT program. Moreover, Eq. 2 had a nonsignificant difference [*t* (44) = 1.238, *P* > 0.05] and lower predictive errors (CE = 0.7 ± 3.7 mL⋅kg^–1^⋅min^–1^, MAPE = 4.8%) than did Eq. 3.

**TABLE 4:** Descriptive examination of the correlations and differences between other indirect methods and the measured VO_2max_.

**Methods**	**Reference**	**Predicted VO_2max_**	**CE**	***t***	***r***	**ICC**	**MAE**	**MAPE (%)**
		**(mL⋅kg^–1^⋅min^–1^)**	**(mL⋅kg^–1^⋅min^–1^)**				**(mL⋅kg^–1^⋅min^–1^)**	
Equation 1	Klusiewicz et al., 2016							
	Male (*n* = 23)	65.7 ± 6.1	5.0 ± 5.6	4.310*	0.568*	0.427*	6.0 ± 4.5	10.3
	Female (*n* = 22)	59.5 ± 5.9	2.8 ± 3.5	3.701*	0.807*	0.723*	3.9 ± 2.3	6.9
	All (*n* = 45)	62.7 ± 6.7	3.9 ± 4.8	5.513*	0.721*	0.604*	5.0 ± 3.8	8.6
Equation 2	Marsh, 2012							
	Male (*n* = 29)	62.1 ± 2.8	-0.2 ± 2.8	-0.333	0.602*	0.600*	2.2 ± 1.7	3.5
	Female (*n* = 16)	57.8 ± 2.6	2.2 ± 4.5	1.954	0.125	0.099	3.6 ± 3.8	6.9
	All (*n* = 45)	60.6 ± 3.4	0.7 ± 3.7	1.238	0.655*	0.615*	2.7 ± 2.5	4.8
Equation 3	Marsh, 2012							
	Male (*n* = 29)	64.0 ± 3.5	1.7 ± 2.3	4.116*	0.787*	0.704*	2.3 ± 1.7	3.6
	Female (*n* = 16)	59.2 ± 3.4	3.6 ± 5.2	2.728*	0.008	0.006	4.8 ± 4.2	9.0
	All (*n* = 45)	62.3 ± 4.2	2.4 ± 3.7	4.352*	0.671*	0.586*	3.1 ± 3.0	5.6

CE, constant error, arithmetic mean of the difference between estimated and measured VO_2max_; *t*, *t* value from paried sample *t*-test; *r*, pearson correlation coefficient; ICC, intraclass correlation coefficient; MAE, mean absolute error; and MAPE, mean absolute percentage error. *Statistically significant (*P* ≤ 0.05). Equation 1: VO_2max_ (mL⋅kg^–1^⋅min^–1^) in males = (3.2131 + 0.0076 × PWC170) / body mass, VO_2max_ (mL⋅kg^–1^⋅min^–1^) in females = (2.4138 + 0. × PWC170) / body mass. Equation 2: VO_2_ (mL⋅kg^–1^⋅min^–1^) = (speed (m⋅min^–1^) × 0.2) + (gradient × speed (m⋅min^–1^) × 0.9) + 3.5, where the estimated maximal speed was calculated as following steps, (1) linear regression was used based on steady-state heart rate values and running speed were obtained for each stage of the submaximal running test, (2) the linear line was then extrapolated to estimated maximal heart rate (220 – age) to determine the value of estimated maximal speed. Equation 3: VO_2max_ (mL⋅kg^–1^⋅min^–1^) = VO_2_-stage 4 + b (HRmax – HR-stage 4), where the VO_2_-stage 4 was calculated based on the steady-state HR in stage 4 of the submaximal running test as the Eq. (2), HRmax refers to estimated maximal heart rate (220 – age), and the additional coefficient b is calculated from b = (VO2-stage 4 – VO2-stage 3) / (HR-stage 4 – HR-stage 3), which is the ratio of the difference between the estimated VO2 of last two stages of submaximal running test and corresponding change of steady-state heart rate.

## Discussion

This study aimed to investigate the accuracy of the FFT method for VO_2max_ estimation using submaximal programs, such as rowing or running, in well-trained athletes. Good levels of agreement between the estimated VO_2max_ from the FFT and the measured VO_2max_ from the GXT and < 10% MAPE were observed in the current study, which met the criteria suggested by Nelson et al. (2016). Additionally, the coefficient of determination (Figure 2) indicated that the FFT method accounted for 69.5% and 75.3% of the variance of VO_2max_ in the ROW and RUN groups, respectively, suggesting that the FFT method estimated VO_2max_ well. Furthermore, the results also illustrated that FFT methods were more accurate in predicting VO_2max_ than the previous predictive equations when using the same submaximal programs in well-trained athletes. These findings indicate that the FFT method can be a fairly accurate option for obtaining VO_2max_ in well-trained athletes. Such submaximal tests can be more widely applied in the sports setting, such as in an individualized training or intervention approach, and during a repeated baseline testing setting.

### Analysis of Estimated VO_2max_ Using the FFT Method

#### Novelty Analysis

Previous studies have developed several equations for predicting VO_2max_ when using submaximal programs in rowing or running exercise (Lakomy and Lakomy, 1993; Vehrs et al., 2007; Akay et al., 2011; Kendall et al., 2012; De Brabandere et al., 2018). Kendall et al. (2012) found an accurate predictive equation [percentage of total error (%TE) = 5.1%, *R*^2^ = 0.707, and %SEE = 4.6%] based on critical velocity and anaerobic rowing test trials. Other studies (Vehrs et al., 2007; Akay et al., 2011) also showed accurate equations for predicting VO_2max_ (*r* = 0.91, SEE = 2.54 mL⋅kg^–1^⋅min^–1^; *r* = 0.94, SEE = 1.80 mL⋅kg^–1^⋅min^–1^) using only the single-stage submaximal treadmill jogging test in healthy adults. However, these equations were developed for the nonathletic population and should be used with caution for well-trained athletes. The findings of this study revealed that the FFT method had a fairly accurate VO_2max_ estimation for well-trained rowers and paddlers and that the FFT method has been proven to accurately estimate VO_2max_ in nonathletic populations (college students, healthy adults, and recreational runners; Kraft and Roberts, 2017; Kraft and Dow, 2019; Passler et al., 2019). However, the rowing exercise program was not included. In the present study, the validity of the FFT method was verified in estimating VO_2max_ in well-trained rowers using a specific submaximal rowing test program.

#### Analysis of Comparison Between the FFT Method and Previous Predictive Equations

In the rowing program, although the results showed that the previous predictive equation (Eq. 1) by Klusiewicz et al. (2016) had a fairly accurate prediction of VO_2max_, the FFT method showed even higher accuracy and lower error terms in all groups, as well as in both the male and female subgroups (Table 4). Interestingly, the estimated VO_2max_ from both the FFT method and Eq. 1 showed a significant overestimation of VO_2max_ (FFT, 1.3 ± 3.5 mL⋅kg^–1^⋅min^–1^; Eq. 1, 3.9 ± 4.8 mL⋅kg^–1^⋅min^–1^). Well-trained athletes have a lower HR at the same intensity than the nonathletic population and have a relatively higher predicted VO_2max_ value based on the linear regression model, which may be the main reason. In addition, the estimation of VO_2max_ from the FFT method in the female subgroup had a nonsignificant difference and a lower MAPE than that in the male subgroup, indicating that the FFT method may provide a more accurate estimation of VO_2max_ in female athletes than that in male athletes when using the rowing program.

In the running program, Marsh (2012) created two equations for estimating VO_2max_ that were suitable for different populations. The present study used these two equations to estimate VO_2max_ in well-trained athletes in the submaximal treadmill running program and found that they all had acceptable accuracy for the overall group. The two equations had similar validity coefficients and agreement levels. However, both equations showed poor accuracy in the female subgroup, which was probably due to the fact that these equations were cross-validated in male athletes instead of in female athletes in the study by Marsh (2012). Similar to the rowing program, the FFT method demonstrated a more accurate prediction of VO_2max_ than these two equations for the overall group. Additionally, the FFT method also had an accurate VO_2max_ estimation in the female subgroup, which was better than that from the two equations. The FFT method modifies equations in the software based on the relevant background variables (e.g., activity class, training zone, and HR variability) and then improves the accuracy of the VO_2max_ estimation, which could explain this phenomenon.

#### Analysis of Estimating Bias From the FFT Method

Sartor et al. (2013) have argued that many HR-based submaximal test programs are known to underestimate VO_2max_ because the workload is not high enough to promote adequate parasympathetic withdrawal and concomitant sympathetic activation. The tendency of underestimation was discovered in previous FFT-related studies (Anderson et al., 2019; Passler et al., 2019). Unlike those findings, this study found that the FFT method overestimated VO_2max_ in both the rowing and running programs. A pilot study to detect suitable workloads in submaximal programs may contribute to this phenomenon. Previous studies have indicated that individualized submaximal testing has been utilized in running (Vesterinen et al., 2017), rowing (Otter et al., 2015), and cycling (Lamberts et al., 2011), and high correlations have been found between power or speed at 90% HR_max_ and maximal endurance performance. Other studies also concluded that an optimal submaximal test program includes a proper target intensity, and different workloads for different characteristics may yield a more accurate prediction (Sartor et al., 2013). This study used workloads that may be close to maximal in some individuals, especially in the running program, which may overestimate VO_2max_.

#### Practical Application of the FFT Method

Taken together, using the FFT method for VO_2max_ estimation has several practical advantages in the evaluation of aerobic capacity in well-trained athletes. First, only a wearable HR device is needed, and the HR data are recorded during the submaximal testing program available in the software; then, the estimated VO_2max_ value with acceptable accuracy would be automatically calculated. Additionally, the FFT method only requires submaximal tests, and multiple athletes can be tested simultaneously, making its use more feasible during the busy training schedule compared to the direct measurement method for VO_2max_ in the laboratory. Thus, the FFT method can be considered as a potentially convenient and cost-effective alternative to measure the maximal aerobic capacity of well-trained athletes, especially for rowing and running.

#### Analysis of Limitations

This study had some limitations. The first one is the lack of information regarding the underlying equation of VO_2max_ estimation for the reason that the exact equation can not be obtained from the company. Second, unlike rowers, the lack of sports event-specific testing (paddling ergometer) in paddlers may limit the applicability of the results. However, the running program was performed in this study for the reason that the achievement of VO_2max_ by treadmill running is consistent with paddling ergometer in well-trained paddlers (Augusto Rodrigues dos Santos et al., 2012). Nonetheless, further studies are needed to develop and investigate the predictive models of the FFT method based on submaximal rowing and running programs, not only in terms of validity but also reliability.

## Conclusion

The results of the present study indicate that the FFT method provides a considerably accurate estimation of VO_2max_ in well-trained rowers, kayakers, and canoeists, which can be considered as a potentially convenient and cost-effective alternative to measure the maximal aerobic capacity of well-trained athletes, especially for rowing and running.

## Data Availability Statement

The original contributions presented in the study are included in the article/Supplementary Material, further inquiries can be directed to the corresponding author/s.

## Ethics Statement

The studies involving human participants were reviewed and approved by Ethics Committee of Zhejiang Institute of Sports Science, Hangzhou, China. Written informed consent to participate in this study was provided by the participants’ legal guardian/next of kin.

## Author Contributions

WG, HF, and QC performed the material preparation, and data collection and analysis. WG and O-PN wrote the draft of the manuscript. WG, O-PN, and XC conducted the revision. XC supervise the whole program. All authors contributed to the conception and design of this study and approved the final manuscript.

## Conflict of Interest

The authors declare that the research was conducted in the absence of any commercial or financial relationships that could be construed as a potential conflict of interest.

## Supplementary Material

Supplementary Table 1 Submaximal FFT programs for both the ROW group and RUN group.

Supplementary Table 2 GXT grograms for both the ROW group and RUN group.
